# Second career of a biosynthetic enzyme: Lumazine synthase as a virus-like nanoparticle in vaccine development

**DOI:** 10.1016/j.btre.2020.e00494

**Published:** 2020-07-06

**Authors:** Rudolf Ladenstein, Ekaterina Morgunova

**Affiliations:** a Karolinska Institutet NEO, Department of Biosciences & Nutrition, Blickågången 16, 14 183 Huddinge, Sweden; b Karolinska Institutet Biomedicum, Department of Medical Biochemistry & Biophysics, Solnavägen 9, 17177 Stockholm, Sweden

**Keywords:** Vaccine development, Lumazine synthase, de novo protein design, Virus-like nanoparticles, Antigen, Immune response

## Abstract

Naturally occurring and computationally *ab initio* designed protein cages can now be considered as extremely suitable materials for new developments in nanotechnology. Via self-assembly from single identical or non-identical protomers large oligomeric particles can be formed. Virus-like particles have today found a number of quite successful applications in the development of new vaccines. Complex chimeric nanoparticles can serve as suitable platforms for the presentation of natural or designed antigens to the immune system of the host. The scaffolds can be cage forming highly symmetric biological macromolecules like lumazine synthase or symmetric self-assembling virus-like particles generated by computational *ab initio* design. Symmetric nanoparticle carriers display a structurally ordered array of immunogens. This feature can lead to a more favorable interaction with B-cell receptors, in comparison to the administration of single recombinant immunogens. Several pre-clinical animal studies and clinical studies have recently pointed out the efficiency of nanoparticle antigens produced recombinantly in creating strong immune responses against infectious diseases like HIV, Malaria, Borrelia, Influenza.

## Highlights

• Virus-like nano-particles can be successfully applied in vaccine development.
• Scaffolds can be cage-forming highly symmetric biological macromolecules, like lumazine synthase, ferritin or self-assembling nanoparticles created computationally *ab initio.*
• Symmetrical nano-particle scaffolds can display structurally ordered immunogen arrays which lead to favorable reaction with B cell receptors.
• Animal-, preclinical- and clinical studies are at present pointing to the usefulness of nanoparticle antigens in creating immune responses against HIV, Borrelia, Influenza.

## 1 Introduction

The immune system has evolved to recognize and respond to nano- and micrometer-sized particles like bacteria and viruses. Nanoparticles are transported to lymphoid tissues, are internalized and processed for antigen presentation by dendritic cells which subsequently leads to the activation of antibody producing B-cells. The strength of the immune answer seems to be dependent on the density (or multivalency) of antigen display by preferably symmetric nanoparticles and the extent of crosslinking B-cell receptors [1]. The detailed mechanisms by which the display of polyvalent antigens on nanoparticles leads to increased immunity are subject to intensive research. Many studies have demonstrated the relation between B cell crosslinking and B cell activation [2]. Both, naturally occurring and computationally designed protein cages can now be considered as extremely suitable materials for new developments in nanotechnology [2]. Via self-assembly from single identical or non-identical protomers large particles can be formed. In many cases their structures have been resolved and their internal symmetries deduced. A crucial advantage is that most of these assemblies can be obtained by heterologous expression of their monomers and that their outer- and inner surfaces as well as their subunit interaction surfaces can be modified by protein engineering methods. Following this way, structural systems with new, even non-natural functions can be created. Naturally occurring protein capsids belong usually to the virus world, are well-studied and several of their structures are used with great advantage for new developments in nanotechnology. As an example, virus-like particles have today found a number of quite successful applications in the development of new vaccines [3]. However, even non-viral protein assemblies, like ferritin [4], bacterial nanoparticles [5] and small heat shock proteins [4] have served as appropriate bases for numerous nanotechnological applications. In this review article we are going to present the capsid-forming bacterial enzyme lumazine synthase and some of its assembly forms as a scaffold for the development of new vaccines. Furthermore, new developments concerning protein scaffolds designed *ab initio* will be discussed.

## 2 Function and multimeric structures of lumazine synthase

**Fig. 1:**
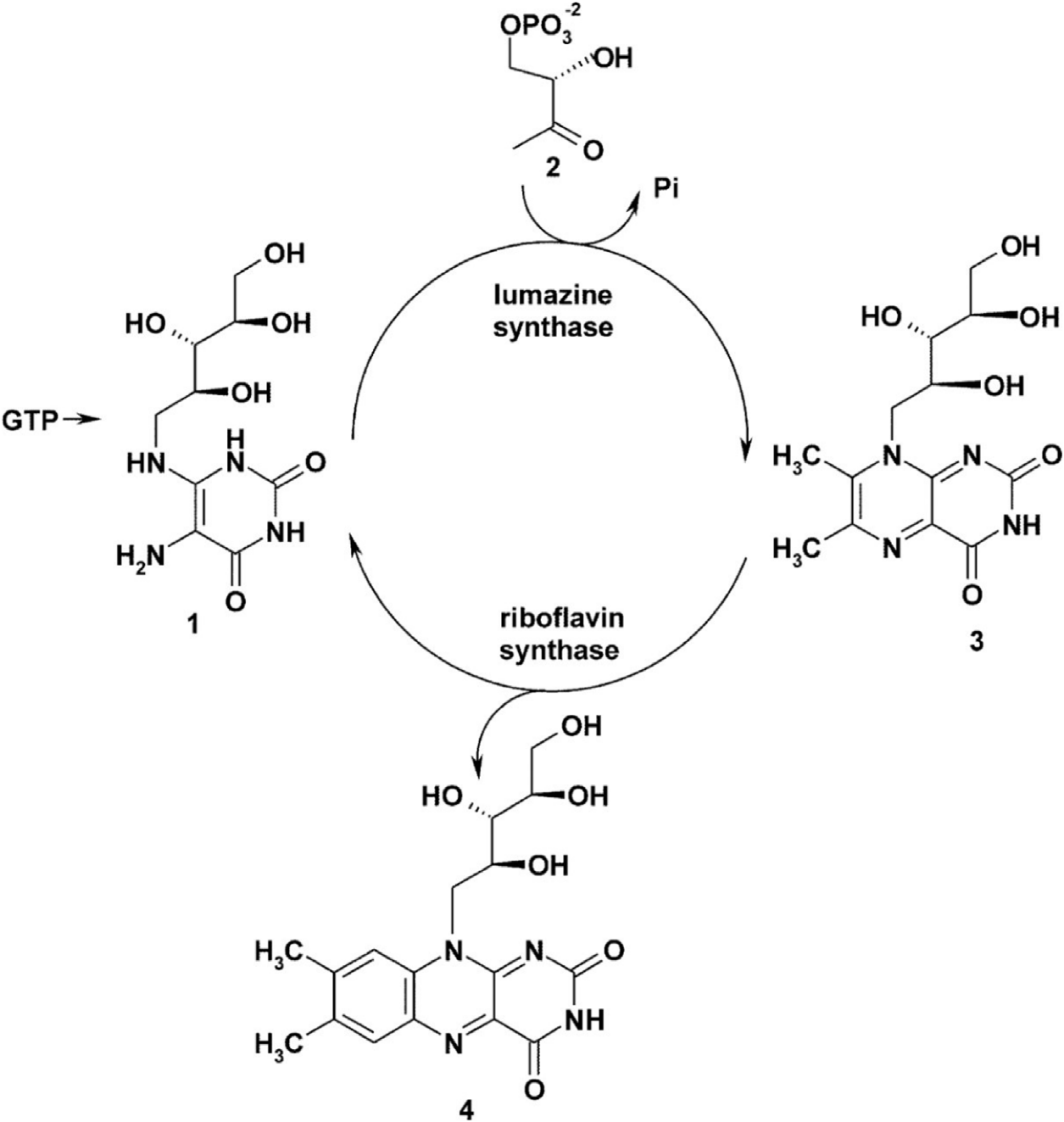
Biosynthesis of lumazine and riboflavin. 5-amino-6-(D-ribitylamino)uracil (**1**), a late stage intermediate, is condensed by lumazine synthase (LS) with l-3,4-dihydroxy-2-butanone phosphate (**2**) to yield 6,7-dimethyl-8-ribityllumazine (**3**). Riboflavine synthase converts 2 molecules of (**3**) to riboflavin (**4**) and one molecule of **1**, which can be re-used by LS in a subsequent reaction cycle.

The enzyme lumazine synthase (LS, EC 2.5.1.78) catalyzes the penultimate step in the biosynthesis of riboflavin, also generally known as vitamin B2. Mammals are strictly dependent on uptake of this vitamin from their diet. Microorganisms and plants produce vitamin B2 in a biosynthetic pathway which starts from GTP [6], see Fig. 1.

**Fig. 2:**
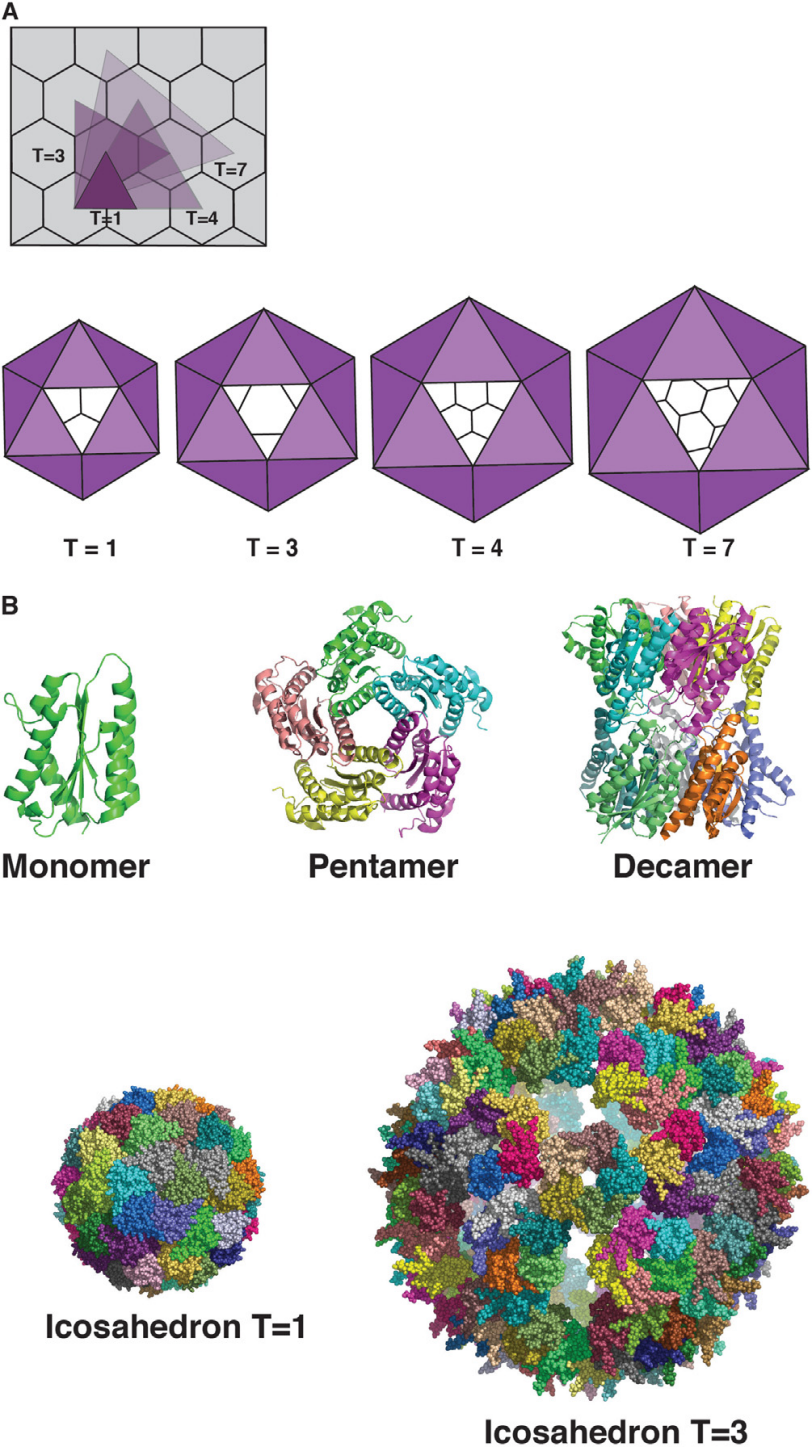
a. Triangulation number of icosahedrons. Generally, an icosahedral structure can be regarded as being constructed from pentamers and hexamers [11]. The structures can be indexed by two integers h, k with h ≥ 1, k ≥ 0; the triangulation number T is defined as T = h^2^ + hk + k^2^; in this scheme icosahedral capsids are built up from 12 pentamers and 10 (T -1) hexamers, the simplest icosahedron with T = 1 contains only 12 pentamers; however, many exceptions to this rule exist in the world of virus structures. Point group symmetry. A regular T = 1 icosahedron has 60 rotational symmetries: 12 fivefold -, 20 threefold -, and 30 twofold vertex points and is described by point group I_h_ (*532); a regular dodecahedron has the same set of symmetries. b. Assembly states of lumazine synthase. The monomer fold of lumazine synthase is shown together with various assembly states, specifically a pentamer, a dimer of pentamers, and icosahedral assemblies comprising 60 monomers (T = 1) and 180 monomers (T = 3). The LS structures are taken from the Protein Data Bank (PDB): Schizosaccharomyces pombe (1KYY), Brucella abortus (1XN1), Aquifex aeolicus (1HQK).

Detailed studies of the reaction mechanism of this enzyme as well as its biochemical function have been described in a number of papers and reviews [6,7] and will not be repeated here. Structural investigations of LS from *Bacillus subtilis* by electron microscopy and X-ray crystallography have been initiated around 1980 [8] and have revealed very interesting and unique – according to the knowledge about enzyme structures at this time – quaternary structures. The enzyme from *Bacillus subtilis* (BsLS) and a number of other bacteria and archaea forms icosahedral capsids with triangulation number T = 1 [[9], [10], [11]]. The capsids have an outer diameter of around 16 nm and are built up by 12 pentameric units, thus consisting in total of 60 identical subunits, which are related by twofold-, threefold- and fivefold symmetry axes. The molecular weight of the icosahedral complex is around 960 000 Daltons. LS’s from fungi and some eubacteria, however, can exist in different quaternary states: either as pentamers [12] or decamers (dimers of pentamers) [13]. Surprisingly, the pentameric riboflavin synthase from Archaea is a paralog of LS [14] (Fig. 2a,b).

Upon buffer exchange from neutral phosphate buffer to an alkaline medium (pH > 8) the BsLS capsid converts from the T = 1 state to a T = 3 capsid consisting of 180 identical subunits with a diameter of around 29 nm [15]. This property is the result of the loss of a phosphate ion per monomer which stabilizes the T = 1 state. Differential calorimetric measurements of LS capsids from *Bacillus anthracis* (BaLS) have shown two major thermal transitions (52.0° and 93.6 °C). The first thermal transition has been interpreted by the thermal dissociation of a phosphate ion from the LS/phosphate complex, the second transition likely corresponds to the dissociation of LS oligomers and unfolding of the monomers [16].

The quaternary assembly modes of the LS capsid structures are similar to those of the capsids of small icosahedral viruses. Structural similarities, however, between LS monomers and capsid proteins from icosahedral viruses have not been detected. The LS monomer rather has characteristical structural similarities to the flavodoxin fold [9].

In its natural environment BsLS encapsulates a riboflavin synthase trimer (BsRS) which catalyzes the final step of riboflavin biosynthesis (see Fig. 1). Several other LS’s are known to form capsid structures [17] but do not necessarily enclose their cognate riboflavin synthase, e.g. by density gradient centrifugation of *Escherichia coli* raw extracts only empty icosahedral LS capsids were found. In 1996 recombinant *Escherichia coli* LS has been isolated as an empty icosahedral T = 1 capsid [18], however, the question whether *Escherichia coli* LS encloses its cognate riboflavin synthase *in vivo* remained unsolved.

Recently, researchers from the ETH Zurich have shown that LS from the archaeon *Aquifex aeolicus* (AaLS) forms inclusion complexes with its cognate riboflavin synthase (AaRS) [19]. Highly interesting, a strongly positively charged 12 amino acid C-terminal peptide from AaRS was found to serve as a localization signal in the recognition process between AaLS and AaRS.

## 3 Assembly control by engineering of capsomer interfaces and -inner surfaces

### 3.1 Studies on interfaces

**Fig. 3:**
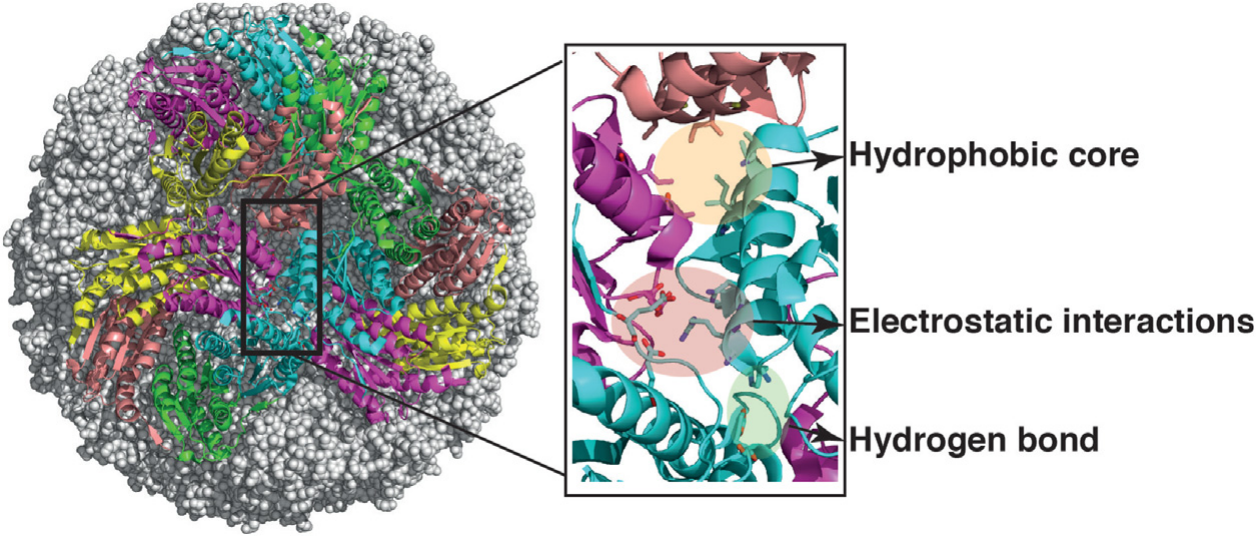
Pentamer-pentamer interactions in Aquifex aeolicus lumazine synthase. Eight residues, comprising a hydrophobic core, a hydrogen bond and a small electrostatic network have an important function in assembly. Mutagenesis can lead to variants that only form pentamers or expanded capsids (see text for more details).

Studies of the interfaces between subunits in virus-like structures can show how their self-assembly depends on specific protein-protein interactions. By engineering of the capsomer interfaces of AaLS, control over the direction of the assembly process was obtained to a certain extent. Structural comparisons of capsid-forming AaLS subunits with those of pentameric *Saccharomyces cerevisiae* LS (ScLS) have led to the hypothesis that a loop at β strand X would prevent the formation of an icosahedral capsid. Following this idea, a four residue sequence, IDEA, from the ScLS loop region was introduced in a homotopical way to AaLS and the variant was designated AaLS-IDEA [10]. The introduction of the IDEA motif, however, was conducive to an unexpected change of the self assembly: instead of the expected pentamers, a capsid with a diameter of around 29 nm was formed, i.e. a T = 3 form, containing 180 identical subunits. Crystal structure analysis of AaLS [10] has revealed 8 amino acid residues, close to the three-fold symmetry axes, forming a hydrophobic array, an electrostatic network and a hydrogen bond, involved in pentamer-pentamer interactions (Fig. 3). The complete disruption of these non-covalent interactions by mutagenesis leads to variants of AaLS that only can form pentameric assemblies [20].

Removal of only the hydrogen bond, or one interaction in the electrostatic network did not hinder the icosahedral assembly. Mutation of Leu121 and Ile125 in the hydrophobic array to Glu, however, has resulted in a variant with expanded capsid (probably T = 3), similar in diameter to AaLS-IDEA (see above).

### 3.2 Mutagenesis of inner capsid surfaces

Surprisingly, even the introduction of negative charges on the inner surface of the LS capsid can lead to changed structural properties which may substantially influence the self-assembly process. Four amino acid residues in AaLS-wt (Arg83, Thr86, Thr120, Gln123) were mutated to Glu residues. The obtained variant was designated AaLS-neg [21]. This variant assembled into hollow capsids with a diameter of around 29 nm, T = 3, and consisting of 180 subunits, bearing 720 interior negative charges. By introduction of even more interior negative charges to AaLS-wt, variants with dramatically changed assembly states were obtained and their structures investigated by mass spectroscopy and cryo EM single particle reconstruction [22]. These studies have shown, supporting the earlier results of Li Xing et al. [23], that the expanded LS capsids did not follow the quasi-equivalence principle which is well-known to provide a morphological basis of a large number of viral capsids [11]. The complexes, designated AaLS-13, assembled into large cages with a diameter of around 40 nm. Large pores were found in their capsid walls. These structures consist of 72 LS pentamers (360 subunits) with icosahedral symmetry. In these highly negatively charged variants the wedge-like shape of the pentamer [9] has changed to a more cylindrical structure producing an increased radius of curvature of the resulting expanded capsid. This feature is beautifully shown in Fig. 3 of reference [2]. It is to be expected that these large icosahedral capsids are more prone to deformation by mechanical forces, however, they have still kept high thermal stability even upon heating at 90 deg C [22]. When the interior capsid surface of AaLS-wt was positively charged by the following mutations (T86R, D90 N, T120R, E122R) giving variant AaLS-pos, 60 subunits assembled into a T = 1 icosahedral capsid [24].

## 4 Lumazine synthase oligomers as display platforms for antigen presentation

A large number of structurally known nanoparticle systems have been chosen in the past and their usefulness for display of polyvalent antigens investigated [25]. Those include inorganic particles, self-assembling protein oligomers and synthetic polymers. Most attractive among them are self-assembling proteins. Synthetic polymers, i.e. *de novo* designed self-assembling protein cages (see section below), however, have gained increased attention recently [26]. Both provide precise size control and can be obtained by recombinant DNA technology. New strategies for the display of polyvalent antigens generate vaccine versions which present multiple copies of an antigen on the highly symmetric surface of a suitable nanoparticle. In this way the surface of a microbial pathogen, e.g. a virus molecule, is mimicked. It has been suggested by several authors that B cells in general react stronger to antigens possessing repetitive, ordered structures. Highly ordered antigens with high local density promote activation of B cell receptors [27] leading to a more efficient B cell activation and proliferation. Display of antigens in a repetitive array on symmetrical virus-like surfaces produced higher antibody levels than those produced by monomeric protein scaffolds [28].

### 4.1 Icosahedral assemblies

Icosahedral LS (60 identical subunits, T = 1, diameter 16 nm) has in recent years become an attractive nanoparticle for display and presentation of polyvalent antigens [16,29,30]. The N- and C- termini of all LS monomer forms are exposed on the surface and point outwards [9,10]. Antigens can be fused to either or both termini by the usual protein engineering techniques. Assembly of the complete icosahedral scaffold is usually not inhibited. The icosahedral LS assemblies consisting of 12 pentameric blocks arranged around the icosahedral five-fold axes show excellent conformational stability. The highest melting temperature, T_m_ = 120 °C, has been measured for LS from *Aquifex aeolicus* (AaLS) [10]. As a promising scaffold LS can form a variety of oligomeric states depending on the species and on appropriate changes in the amino acid sequence: pentamer, decamer, 60-mer (T = 1 icosahedron), 180-mer (T = 3 icosahedron), 240-mer (T = 4 icosahedron) and probably larger cages.

The location of the N- and C-terminus on the surface of the lumazine synthase capsids from *B. subtilis* and *A. aeolicus* prompted concepts to use gene engineering technology in order to decorate the surface of lumazine synthases with multiple copies of peptide motifs or protein domains. With the explicit aim to generate a basis for future vaccine development, the feasibility of this concept was explored with LS of *B. subtilis* and with the hyperthermostable LS of *A. aeolicus*. Attaching a variety of peptide motifs to the C-terminus, N-terminus or both termini was tolerated without any impact on the association behavior of the subunits. Recombinant T = 1 icosahedra comprising 60 subunits were invariably formed. As a specific example, the covalent decoration of LS with oligohistidine tags were shown to enable the cross-linking of metal-binding proteins [71].

Even if BaLS could be proven as a suitable scaffold for vaccine development, the design of the immunogenic chimera has to occur in a way that the antigen to be presented remains in its most immunologically relevant structure. The intrinsic immunogenicity of LS, which is a bacterial protein, represents an important feature which cannot be neglected. Carrier-induced suppression of epitopes may reduce the desired immune response because of preexisting immunity to the carrier protein [32]. The most suitable scaffold should have negligible intrinsic immunogenicity. Solutions to the problem may be found through changes of the carrier surface by computationally designed point mutations [32]. Another possibility, the conjugation with glycans to mask the LS scaffold is described in this review. However, if the antigen in question would be large enough to cover significant parts of the LS surface, proper masking of the immunogenic effect of the LS scaffold could be achieved.

### 4.2 Decameric assembly

The group around Fernando Goldbaum, Buenos Aires, Argentina, has in a number of studies shown the suitability of decameric LS from *Brucella spp (*BspLS) as a vaccine presentation system. In fact, BspLS was the first LS to be explored in this way [34]. BspLS comprises a dimer of pentamers in head-to-head arrangement (see Fig. 2), as such it represents a highly stable scaffold. To the 10 N-termini of the complex, in fact 5 at each pentamer, immunogenic peptides can be fused without detectable changes in folding or stability of the carrier protein. Several examples of LS nanoparticle-based vaccine candidates are listed in Table 1.

**Table 1:** Examples of LS nanoparticle-based vaccine candidates.

Platform	Antigen	Target	Expression system	Reference
Icosahedral assemblies				
AaLS, T = 1	OspA polypeptide	*Borrelia burgdorferi* *Borrelia bavariensis*	*Escherichia coli*	[72]
BaLS, T = 1	PB10 peptide	Ricin Toxin	*Escherichia coli*	[16,32]
AaLS, T = 1	OT-1 and OT-2*	antigen delivery to dendritic cells (DC)	*Escherichia coli*	[33]
Decameric Assemblies				
BspLS	Omp31**	*Brucella abortus*	*Escherichia coli*	[35,36]
	Omp31	*Brucella ovis*		
BspLS-Omp31 chimera	Omp31	*Brucella ovis*	via encoding DNA vaccine	[37]
BspLS	L7/L12 ribosomal protein	*Brucella suis*	*Escherichia coli*	[38]
BspLS	Stx2B Shiga toxin type 2	enterohemorrhagic *Escherichia coli* (EHEC)	*Escherichia coli*	[39,40]
BspLS	OT 257-264 immunogenic T-cell epitope	cancer	*Escherichia coli*	[41]

* OT- Ovalbumin peptide.

** Omp - Outer membrane protein.

## 5 Glycosylation as a design parameter for the development of nanoparticle vaccines

Recent research [43], may have a strong influence on the development of new principles for vaccine design. It has been shown that engineering of nanoparticles with glycans produces increased antibody responses and may drive the development of a new generation of vaccines against widely diverse pathogens, like HIV, tuberculosis and malaria.

**Fig. 4:**
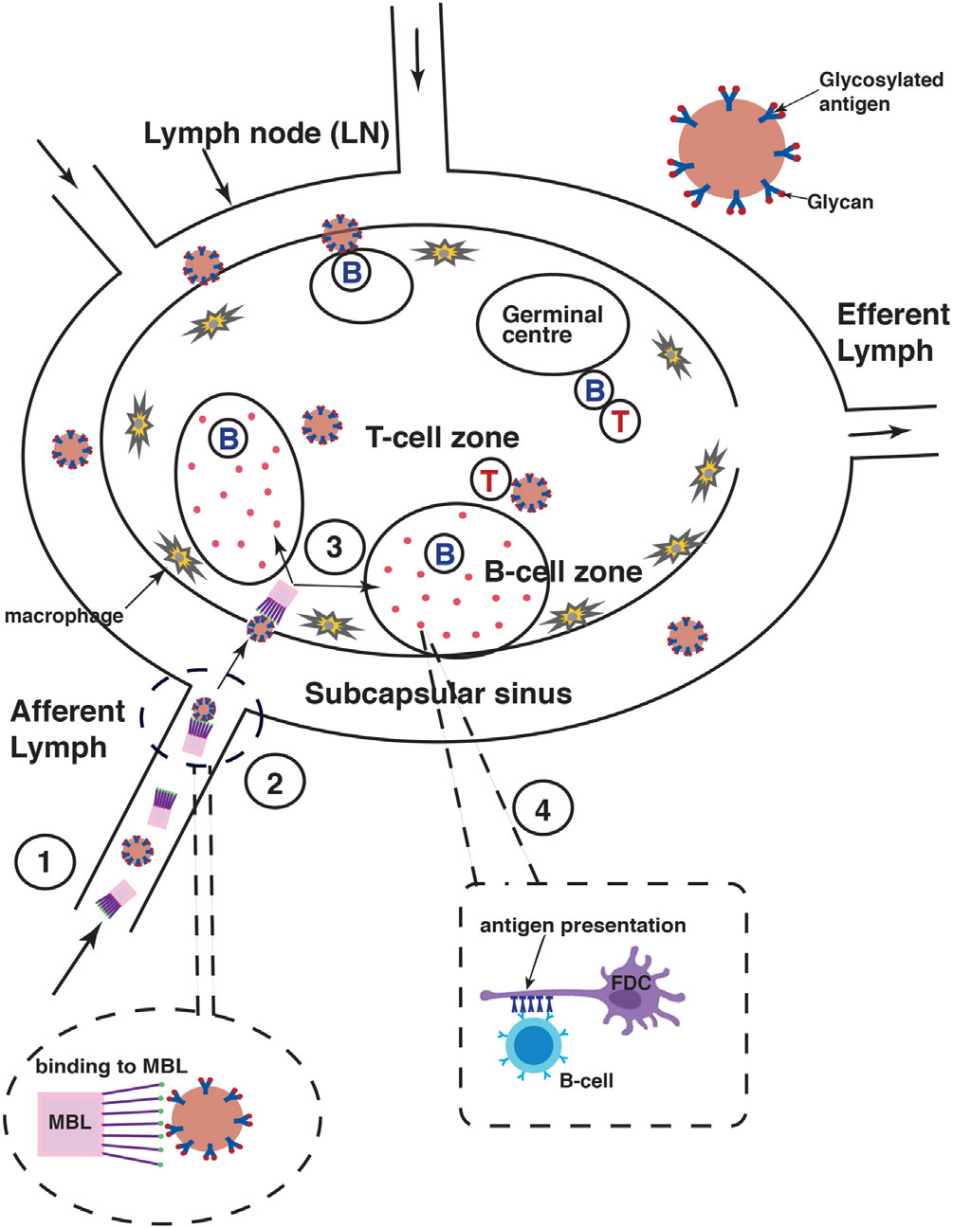
Facilitated nanoparticle transport in lymphatic vesicles (simplified). **(1)** Nanoparticle vaccines may drain into lymphatic vesicles subsequently to i.m. vaccination **(2)** Nanoparticles engineered with glycan arrays are recognized more easily by mannose binding lectin (MBL) [45,69] and are less prone to become trapped by macrophages residing in the subcapsular sinus zone. **(3)** Recognition by MBL facilitates location to compartments called B cell zones **(4)** In the B cell zones (lymphoid follicles) follicular dendritic cells (FDC) present antigens to B cells in their neighborhood. This leads to so-called affinity maturation, which is a selective process that produces high affinity antibodies.

Vaccine design generally starts with the demand that the antigen at least must reach one of (in humans) several hundreds of lymph nodes which are known to represent the control centers responsible for an immune answer. Nanoparticles in a size range of around 10–100 nm in diameter show a great potential to be effective for vaccine design because they have an ability to drain from interstitial tissue into lymphatic vessels. This may result in their accumulation within local lymph nodes [43]. Antigens can be trapped on macrophages in the subcapsular sinus proximal to the B cell follicle. However, B cells at this anatomical site are able to access even trapped antigens. The key to induce immune responses is efficient trafficking to lymphoid tissue [44] (Fig. 4).

Tokatlian et al. [45] have demonstrated that glycosylated nanoparticles presenting a dense array of glycans at their surface can be recognized by mannose-binding lectin (MBL) of the complement system. MBL binds, among several others, mannose glycans which facilitate the delivery and transport of the particles to the B cell locations and induce a cellular cascade including antigen presentation by follicular dendritic cells (FDC) and subsequent concentration in germinal centers *in vivo*. This process finally leads to an enhancement of the immune answer. Bare symmetric ferritin nanoparticles without surface glycosylation showed low accumulation in lymph nodes and colocalization with FDC’s after immunization of mice [45]. Conjugation of ferritin particles with a synthetic trimannose glycan led to strong colocalization on FDC’s after 3 days of immunization. And, most interestingly, the observed colocalization turned out to be dependent on the density of glycans on the ferritin surface. A lower surface density of the trimannose groups (around 25 versus 96 groups/particle) did not result in colocalization with the FDC moieties.

Besides the work on ferritin particles it could also be shown with the eOD-60-mer particle, which is based on *Aquifex aeolicus* lumazine synthase (see sections 6.1 and 6.2), that glycosylation obviously supports trafficking to B cell locations: WT mice immunized with nanoparticle eOD-60 mer showed an IgG response two times that elicited by de-glycosylated eOD-60 mer.

To assess the effects of particle size the same group [45] loaded monodisperse styrene nanoparticles with trimannose groups at high surface density. A distinct size effect could be observed: polystyrene particles with 40 nm diameter collocated with the FDC’s whereas particles with 100–200 nm in diameter did not accumulate in the lymph follicles.

Synthetic introduction of glycans, genetically or chemically, into nanoparticles of the proper size facilitates the direction and complement dependent transport of the particles to the FDC network and the subsequent concentration in germinal centers. Clearly, a dependence on particle size and surface glycan density can be listed as an important design criterium for the development of nanoparticle vaccines. Advances in the synthesis of complex glycans [42] together with a controlled display on nanoparticles may lead to an improved generation of vaccines.

### 5.1 The icosahedral lumazine synthase cage – a scaffold for the promising anti-HIV vaccine nanoparticles eOD-GT6-60mer and eOD-GT8-60mer

HIV infections are a major health problem worldwide. The lack of an effective vaccine causes an enormous medical need, due to about 35 million people worldwide carrying the virus (and may distribute the virus further) and a morbidity of 1.7 million people per year (AVERT aids statistics). A main difficulty in designing an anti-HIV vaccine is the high mutation rate of the virus. A large part of the exposed surface of the envelope glycoprotein (Env) of HIV viruses (true also for influenza- and hepatitis C virus) are hypervariable or shielded by glycans [46]. Furthermore, because of safety measures in the case of HIV, it appears dangerous to administer attenuated or killed virus. An additional problem, as discussed by Schief et al. [46], is the low recognition power of germline precursors of broadly neutralizing antibodies (bnAbs), for example those belonging to the VRC01 class, against wt gp120 the single immunogenic glycoprotein present on the surface of the HIV virus envelope. Thus wt gp120 does not elicit the necessary neutralizing antibodies, however, has since long been considered to be a promising target for the development of a HIV vaccine, because it can bind to the CD4 receptor [47], a protein that is expressed and located on the surface of all T-helper cells.

In a first line, structure-based insights [48] and new methodological approaches have paved the way to what is today known as modern vaccinology [49]. A genome based approach to vaccine development leading to the birth of so-called “reverse vaccinology” [50] came with the sequencing of the *Haemophilus influenzae* genome in 1995 [51]. Vaccine development further moved forward with the gain of knowledge which allowed to rapidly identify highly immunogenic antigens by analyzing the human immune system at the level of single cells and specific proteins, leading to an eventual assessment of the antibody response [52]. The production of germ-line (GT) targeting antigens for vaccine design represents an important example of modern vaccine development [49]. In case of a highly variable virus, such as HIV, the use and development of GT antigens can lead to the production of bnAbs which should be able to clear multiple infective virus strains [3]. Development of vaccines based on GT targeting antigens has been successfully applied to elicit a specific class of gp120 CD4-binding site specific bnAbs of the HIV-1 virus (known as VRC01, see also [53]) by using **e**ngineered **O**uter **D**omain **G**ermline-**T**argeting (**eOD-GT**) peptides [29].

**Fig. 5:**
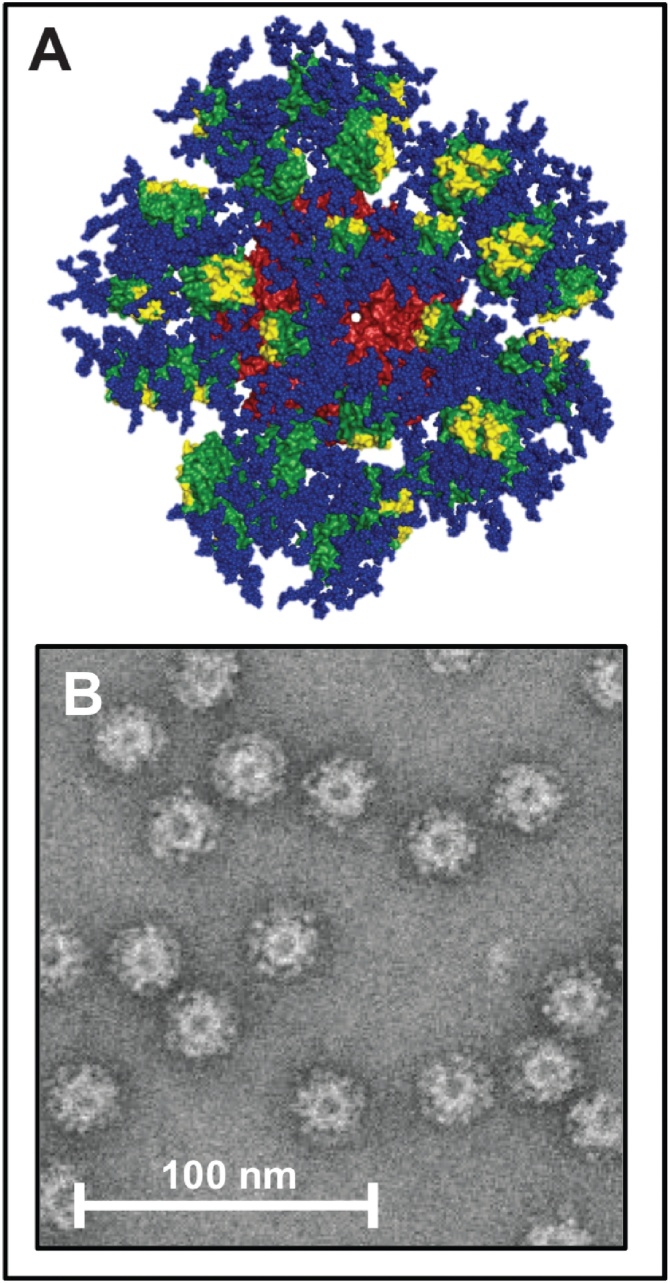
Computer-designed model of the eOD-GT6 nanoparticle. **A** eOD-GT6: green spheres, residues that interact with CD4: yellow spheres, Glycans: blue spheres, icosahedral 60-mer *Aquifex aeolicus* lumazine synthase to which eOD-GT6 is fused: red **B** Negative stain electron microscopic image of the 60-mer eOD-GT6 nanoparticles. From: Joseph Jardine et al., “Rational HIV immunogen design to target specific germline B cell receptors”, Science 2013, 340, 711−716. Reprinted with permission from AAAS.

An approach to overcome several obstacles discussed above by relying on a virus-like particle (VLP) was recently reported by Schief and colleagues [29]. They were able to boost the affinity of the germline antibodies for the gp120 glycoprotein by fusing multiple copies (in fact 60) of an engineered version of gp 120 to the icosahedral 60 subunit complex of lumazine synthase (LS). More specifically, the thermostable AaLS was used as nanoparticle platform for the display of the epitope [10]. It is well-known that the envelope glycoprotein (Env) is the only HIV surface protein to which neutralizing antibodies bind. Env is made of three gp160 precursor proteins that form a trimer. Each of them is subsequently cleaved into gp120 and gp41 monomers. Jardine et al. [29] engineered an optimized subcomponent of the wt gp120 antigen, termed eOD-GT6, which showed excellent binding with dissociation constants (K_D_) in the nanomolar range to diverse GL antibodies. The optimized antigen was fused with a suitable linker to the AaLS gene construct. Recombinant nanoparticle antigens were obtained from mammalian cell cultures and purified by lectin chromatography in good yield, resulting in self-assemblies of AaLS 60 mers (eOD-GT6-60mer), on its surface displaying glycosylated eOD-GT6 peptides, Fig. 5. The eOD-GT6 antigens were sterically positioned in an orientation that would expose the VRC01 epitope.

Monomeric eOD-GT6 did not lead to an activation of B-cells, as shown by Ca^2+^-dependent activation assays. In contrast, eOD-GT6-60mer nanoparticles did activate both, germline- and mature B-cells. Trimeric eOD-GT6 activated both GL and mature B cells, but less potently and less rapidly than the 60 mers. A soluble gp140 trimer from a HIV-1 strain showed no activation of B cells but activated the mature counterparts. The resulting immunogen eOD-GT6-60mer appeared as a promising candidate for further development of a HIV vaccine. In particular its ability to display a symmetric antigen array with spherical shape may lead to favourable cross-linking of B-cell receptors [54].

Screening, sequence analysis and structural investigations by X-ray crystallography have led to further improvement of the eOD-GT6-60mer. The most promising immunogenic candidate is the self-assembling nanoparticle eOD-GT8-60mer based on a fusion of 60 copies of a sequence-modified gp120 glycoprotein with the icosahedral complex of AaLS, which has been mutated on three crucial positions to eradicate the enzymatic activity of the scaffold [30].

In an animal model study using rhesus monkeys, antigen-specific germinal center responses occurred rapidly after a single immunization with the eOD-GT-8-60mer nanoparticle [55]. It was also shown that the way how the vaccine candidate was administered had a strong impact on the immune response in local lymph nodes. More specifically, by using fine needle aspirates of draining lymph nodes, the kinetics of primary immune responses in rhesus monkeys were followed which were immunized i.m. or s.c. with the eOD-GT-8-60mer nanoparticle immunogen in order to facilitate the design of clinical trials. Significant numbers of germinal center B-cells and antigen-specific CD4 T-cells could be detected in draining lymph nodes 7 days after immunization, with a peak reached at day 21. A surprising and important result was that s.c. immunization gave 10 fold larger antigen-specific germinal center B-cell responses in comparison to i.m. administration [56]. This study indicated that antigen-specific germinal center response can occur rapidly after a single immunization with a nanoparticle immunogen.

The chimera eOD-GT8-60mer has shown high affinity and binding breadth to germline-reverted VRC01-like bnAbs [57]. It is presently under investigation in a phase I clinical trial in healthy adults to test safety, tolerability and immunogenicity (NCT 03547245).

### 5.2 Self-assembling HIV Immunogen nanoparticles encoded by RNA replicons

RNA replicons have a strong potential to serve as promising platforms for vaccine development [58]. Quite recently an alphavirus replicon which expresses the self-assembling nanoparticle immunogen eOD-GT8-60mer was designed and tested [59]. Replicon RNA (1 replicon RNA molecule/particle) was encapsulated in lipid nanoparticles, consisting of PEGylated and cationic lipids, that gave effective delivery in mouse muscle, in comparison to low expression levels obtained by delivery of equivalent modified mRNA. Replicons encoding eOD-GT8-60mer led to high titers of gp120-specific antibodies after a single injection in mice. Compared to direct recombinant protein immunization RNA replicon vaccination elicited 2-fold greater levels of antigen-specific germinal center B-cells. Germline targeting (GT) approaches are intended to proceed with multiple immunogens. Vaccination strategies with cocktails of multiple antigens would be expensive and complex for mass vaccination. However, the possibility to use synthetic nucleic acids could facilitate clinical application of these concepts. The study by Melo et al. favours replicon delivery of Env immunogens as a practicable route for HIV vaccine development. The method will presumably be of great value for vaccine development in a more general sense.

## 6 OUTLOOK: de novo design of highly symmetric self-assembling protein nanocages

Above we have shown and discussed the possibilities which are offered by the natural enzyme complex lumazine synthase and its different assembly forms to serve as a scaffold for modern vaccine development. However, as protein science has not stood still in the past years, advances have been made in the design of self-assembling biomaterials. Particularly interesting to mention in this context are new developments concerning protein cages. Any self-assembling multimeric structure must possess certain kinds of interactions between its monomers which are able to energetically drive the assembly process. Complex protein assemblies are usually held together by a large number of weak, non-covalent forces which can form complementary low-energy protein-protein interfaces [60]. The *de novo* design of protein assemblies with such properties has been hindered by a number of obstacles, among them the computational modelling of protein structure and –energetics. Recent developments, however, include the design of homo-dimeric and hetero-dimeric protein interfaces [61,62].

### 6.1 Ab initio design of nanoparticles

King et al. [26] have developed a general computational approach for the design of self-assembling protein nanocages. The procedure includes the following main steps:

1 A target symmetric architecture has to be chosen, (e.g. octahedral point group symmetry with four threefold rotation axes is a possibility)
2 Symmetric oligomers (e.g. C3 symmetric trimers from PDB) which share a symmetry element with the target structure are selected as capsomer building blocks
3 Multiple copies of the building block are symmetrically arranged and aligned with the symmetry axes of the target structure; the active organization of the building blocks fixes several rigid body degrees of freedom, however, two of them remain, a radial shift **r** and an axial rotation **ω**
4 Symmetrical docking of the building blocks is performed by varying the two remaining degrees of freedom **r**, **ω** systematically; the suitability of each configuration for interface design is estimated by a computational criterion which uses interface size and surface complementarity
5 Sequence design calculations (RosettaDesign, [63]) are performed to create low energy protein-protein interfaces between the building blocks that can drive self-assembly.

**Fig. 6:**
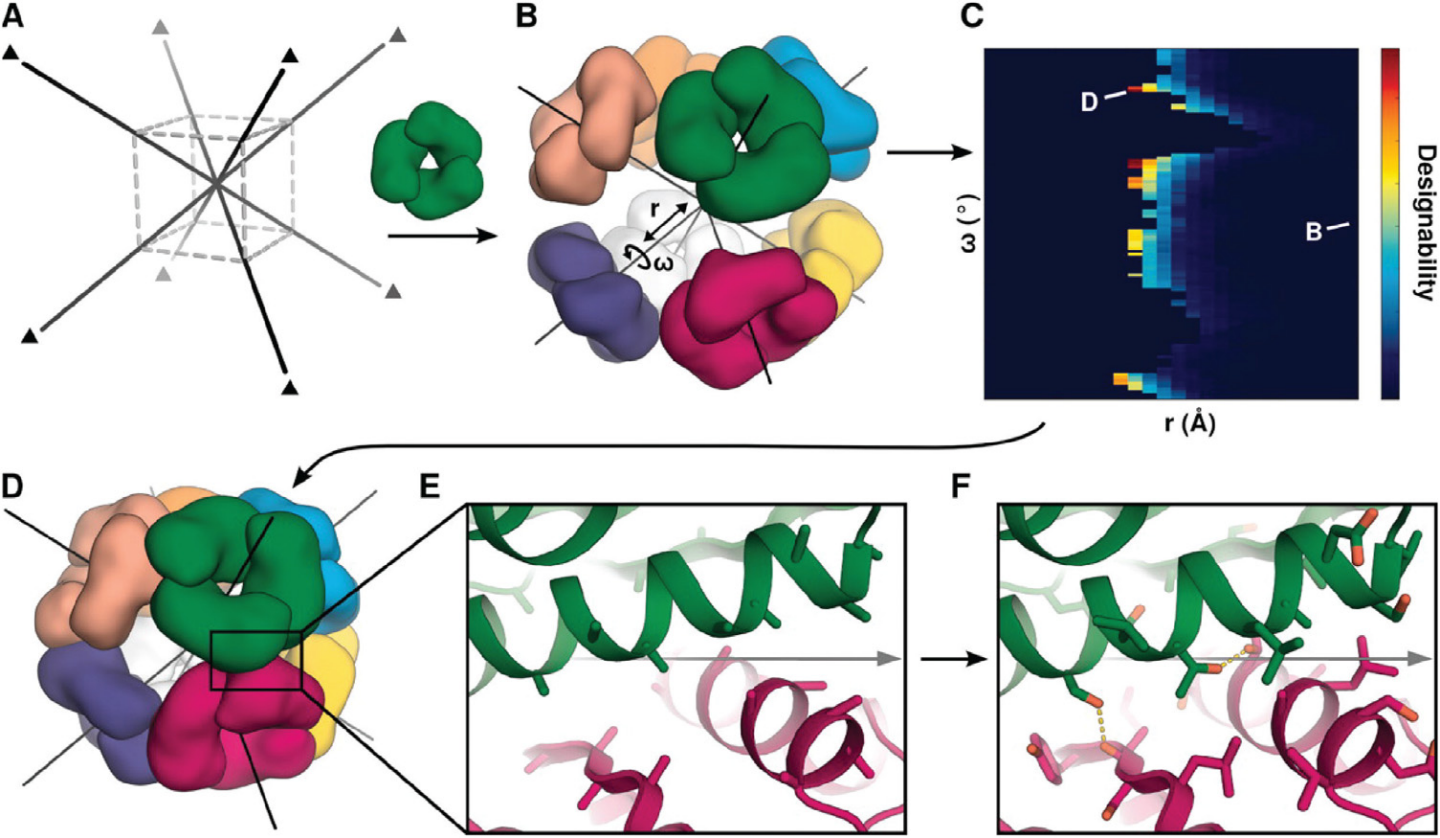
Design of self-assembling protein cages. **A,** Selection of a symmetric target architecture, octahedral point group symmetry is used here. **B,** multiple copies of a building block (a C3 symmetric trimer in this case) are symmetrically arranged in the target architecture, followed by symmetrical docking and varying the two remaining rigid body degrees of freedom (see text). After computing the suitability **(C)** of each configuration for interface design a low energy protein-protein interface is created, that is able to drive self-assembly **D, E, F.** From: Neil P. King et al., “Computational design of self-assembling protein nanomaterials with atomic level accuracy”, Science 2012, 336, 1171−1174. Reprinted with permission from AAAS.

Designs with the lowest predicted interaction energies of the adjacent oligomers (e.g. trimers) were further optimized in RosettaDesign [63] and used for genetic engineering and recombinant expression of the respective symmetric protein complexes. Crystal structures of the obtained assemblies were solved to high resolution. Together with cryo EM reconstructions at low resolution they confirmed the designed protein assemblies remarkably well, e.g. the backbone RMSD reported of an octahedral assembly (designated O3-33, diameter about 13 nm) over all 24 peptide chains was 1.07 Å (Fig. 6).

In a similar manner as described above a 60 subunit T = 1 hyperstable icosahedron was designed [64]. As target architecture icosahedral point group symmetry containing two-, three-, and five-fold rotation axes was chosen and selected trimeric protein scaffolds of known structure (PDB) were arranged with icosahedral symmetry (trimerp axes aligned with the three-fold icosahedral axes). One designed structure, designated I3-01, was purified using immobilized metal affinity and size exclusion chromatography. The structure of this particle was studied using cryo-electron microscopy showing the overall icosahedral architecture clearly. A 3D structure model calculated from the cryo-EM data matched the I3-01 design model well with a correlation coefficient of 0.92 at 20 Å. The icosahedral particle (diameter around 25 nm) is extremely stable: at up to 80 °C in 6.7 M guanidine hydrochloride. It undergoes abrupt, but fully reversible disassembly between 2.0–2.25 M guanidinium thiocyanate. It was suggested that this property may have some importance for cargo packaging.

### 6.2 *De novo* design of a self-assembling vaccine candidate for respiratory syncytial virus (RSV)

**Fig. 7:**
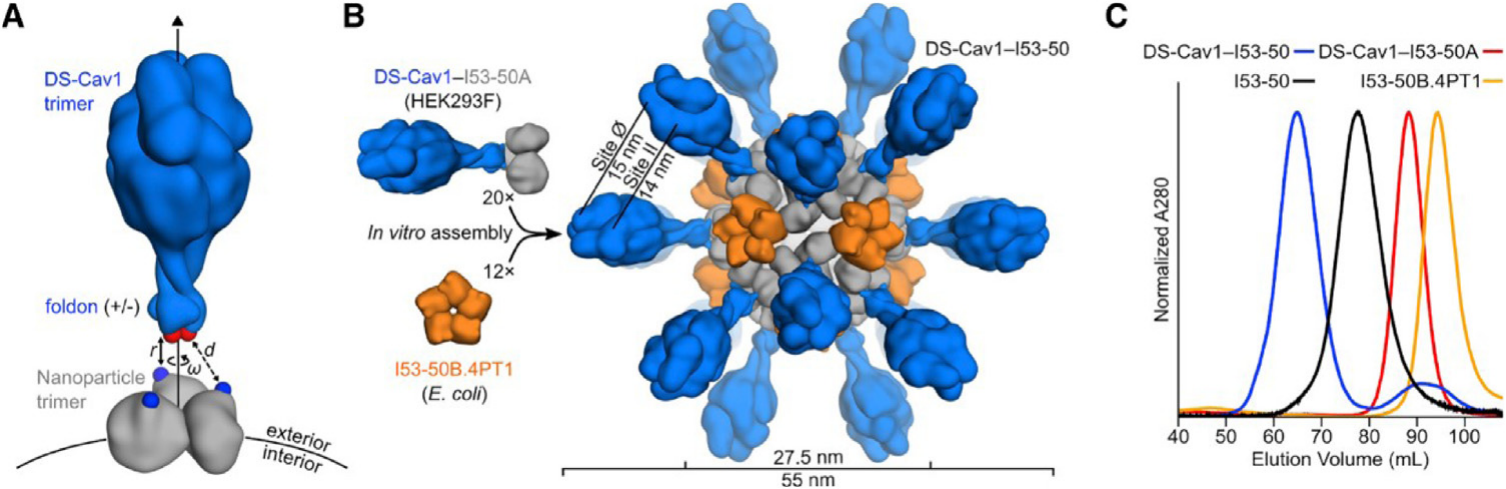
Design and assembly of the nanoparticle designated DS-Cav1-I53-50. **A,** Nanoparticle components which form DS-Cav1 by fusion. Red and blue spheres depict C-termini of the foldon and N-termini of the nanoparticle, respectively. Exterior and interior surfaces of the nanoparticle are indicated. **B,** Structural model of DS-Cav1-I53-50. The in vitro assembly is shown schematically. The assembled nanoparticle comprises 60 copies of each subunit, and is built up by 20 trimeric and 12 pentameric building blocks. **C,** Chromatograms of unassembled components and assembled nanoparticles. Column: Sephacryl S-500 HR 16/60 SEC From: Marcandalli et al.;” Induction of potent neutralizing antibody responses by a designed protein nanoparticle vaccine for respiratory syncytial virus”, (2019) Cell 176(6), 1420-1431 (open access article),

In a remarkable tour de force Marcandalli et al. [65] have designed a self-assembling protein nanoparticle with a fused variant of the F glycoprotein trimer DS-Cav1 of respiratory syncytial virus (RSV). RSV is an enveloped RNA virus in the pneumoviridae family, causing diseases of the respiratory system in children and adults. The nanoparticle scaffold, made up of two components enabled the production of immunogens that displayed the trimeric DS-Cav1 in a symmetrical array on the exterior surface of the nanoparticle. In more detail, the two-component complexes with icosahedral symmetry (T = 1) present 20 copies of the DS-Cav1 trimer, one trimer along each pole of the 10 icosahedral 3-fold symmetry axes (see Fig. 7).

By the new vaccine candidate a 10-fold gain in neutralizing antibodies has been achieved due to structure-based stabilization of the pre-fusion trimer (PreF) [66] and an additional 10-fold increase added by the fusion of the antigen to the nanoparticle, for a total 100-fold increase in neutralizing antibodies relative to the native soluble F trimer (PostF). This promising vaccine candidate has proven that *ab initio* designed two-component nanoparticles may serve as robust scaffolds for structure-based vaccine design. The superior immunogenicity of antigens exposed on symmetrical nanoparticles, as compared to single soluble antigens, is dicussed in sections 6.1 and 8.

### 6.3 The oligomer fusion approach

A different approach, the oligomer fusion approach [67], works entirely with natural protein-protein interfaces and avoids the problem of designing new interfaces. A fusion of two different oligomeric proteins which will be arranged in a particular orientation is created: e.g. fusing a dimeric to a trimeric protein domain (taken from PDB) brings together two symmetry axes. Different overall architectures are possible depending on the relative geometric arrangement of the two symmetry axes. Large, highly symmetric assemblies can be created by their repeated application.

**Fig. 8:**
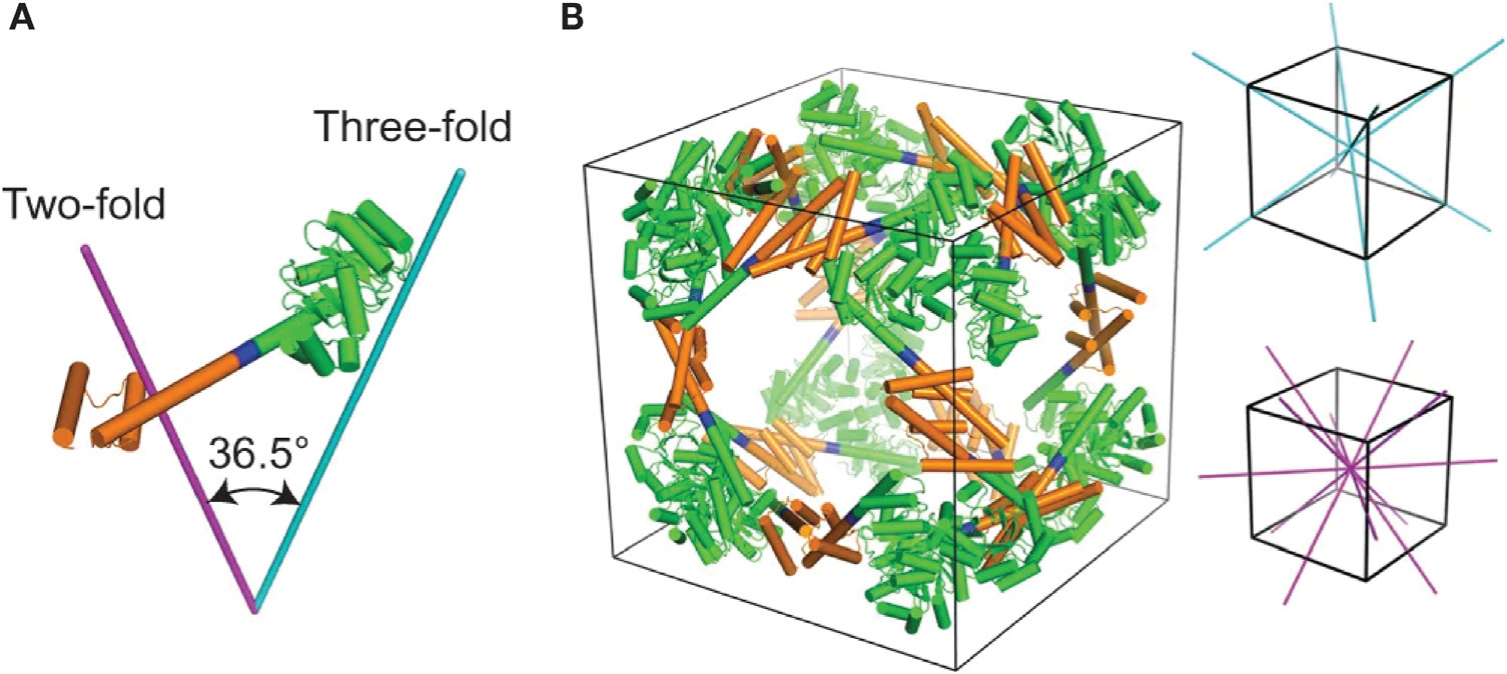
Model of an engineered fusion protein and its assembled protein cage. **(a),** Designed fusion protein with trimeric KDPGal Aldolase (green), the helical linker (blue), and the dimeric domain of the *E. coli* FkpA protein (orange), shown together with the 3-fold (cyan) and 2-fold (magenta) symmetry axes, separated by an angle of 36.5 deg (angle between a face- and a body diagonal of a cube). **(b),** model of the designed 24 subunit cage with octahedral symmetry in a cubic box. On the right: the threefold- (cyan) and twofold (magenta) symmetry axes of a cube are shown. From: Yen-Ting Lai et al., “Structure of a designed protein cage that self-assembles into a highly porous cube “, Nature Chemistry 2014, 6, 1065−1071. Reprinted with permission from Springer Nature.

Specifically, to design a protein assembly with cubic (octahedral) symmetry Lai et al. [67] have employed the so-called helix-based oligomer fusion strategy [68]: to orient the separate oligomeric domains (which are required to have alpha-helical termini) in a defined manner, a short alpha-helical linker is used to join both components. In order to form a 24-subunit cube when joined end-wise by the linker, their 2- and 3-fold symmetry axes have to intersect at an angle close to 35.3° (angle between a face diagonal and a body diagonal of a cube). The cage structure (designated ATC-HL3 cage, 24 subunits) which was finally obtained, showed good agreement with the designed model in a crystal structure analysis. Electron microscopy, mass spectroscopy, and small-angle X-ray scattering, however, pointed to a mixture of alternative assembly forms occurring in solution, 12-mer, 18-mer, 24 mer. The highly porous cage structure ATC-HL3 has a diameter of 22.5 nm with large pores of 10 nm diameter (see Fig. 8).

## 7 Concluding remarks

**Fig. 9:**
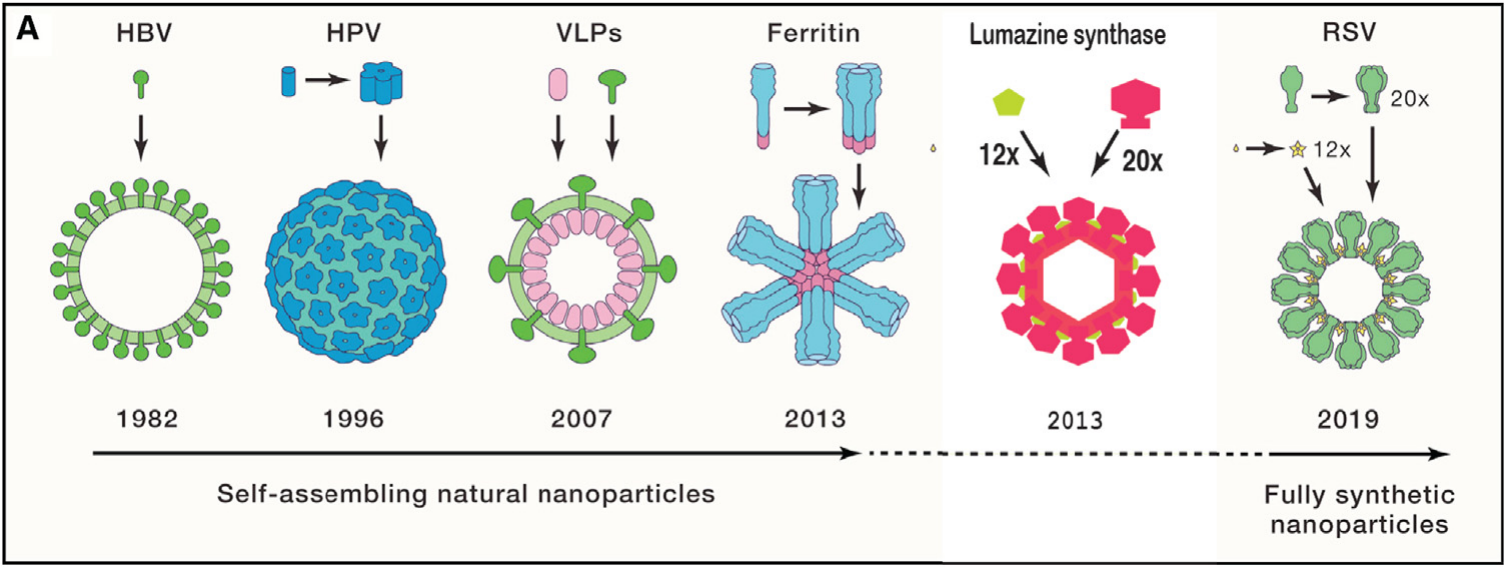
The evolution of nanoparticles in vaccine development over time. HBV, hepatitis B virus; HPV, human papilloma virus; VLP, generic virus-like particle (*Meningococcus* B vaccine); Ferritin, nanoparticles with influenza virus trimeric spike proteins; Lumazine synthase, icosahedral *Aquifex aeolicus* LS cage displaying eOD-GT6 trimers (HIV virus), see section 6.1; RSV, *ab initio* designed icosahedral nanoparticle exposing 20 copies of the DS-Cav1 trimer of respiratory syncytial virus, see section 7.2; (Figure taken and modified from R. Rappuoli and D. Serruto, “Self-Assembling Nanoparticle Usher in a New Era of Vaccine Design”, (2019) Cell 176, march 7, p. 1247).

In this work we have reviewed how virus-like nanoparticles can provide solutions in the field of vaccinology. Complex chimeric nanoparticles can serve as suitable platforms for the presentation of natural or designed antigens to the immune system of the host. The scaffolds can be synthetic polypeptides, cage forming highly symmetric biological macromolecules like ferritin or lumazine synthase, or symmetric self-assembling virus-like particles generated by computational *ab initio* design. Fig. 9 shows a time-line of nano-structures involved in vaccine development, starting from the empirical approaches of the late 1970s via natural scaffolds to full *ab initio* design of protein-based vaccine molecules. On the background of the immense progress in protein crystallography, electron microscopy, computational protein design and, last but not least, recombinant DNA technology finally a completely rational design

of vaccine molecules has become reality.

As a consequence of the rapidly expanding capabilities of computational protein design, development of structure-based approaches to create self-assembling immunogens will help to improve potency, stability and breadth of vaccines against several important pathogens. In comparison to the more-or-less fixed structural parameters of natural scaffolds will the *ab initio* design of self-assembling protein complexes with atomic-level accuracy enable the creation of vaccine candidates with specifically tailored structural features [26,64]. At the building block level multi-component systems with matching symmetry properties can be selected. At the level of the assembled nanomaterial self-assembling scaffolds can be created that exhibit maximum antigen density [65]. Further optimization of these features may allow precise and systematic variation of structural determinants such as size of the immunogen and definition of spacing in immunogen arrays.

For some bacterial proteins used as scaffolds the intrinsic immunogenicity of the carrier might be non-negligible. There are, however, effective design methods available to provide shielding of intrinsically immunogenic carrier surfaces [68,69]. Symmetric nanoparticle carriers display a structurally ordered array of immunogens. It has been shown repeatedly that this feature can lead to a more favorable interaction with B-cell receptors, in comparison to the administration of single recombinant immunogens. Several pre-clinical animal studies and clinical studies have recently pointed out the efficiency of nanoparticle antigens in creating strong immune responses. The design of chimeric nanoparticle antigens, either on the basis of natural virus-like protein scaffolds or by protein assemblies designed by computational ab initio methods appear to find its place in vaccine development at present as these systems can be produced recombinantly in prokaryotic or eukaryotic cell cultures, and thus may enable the production of safe and effective vaccines against a number of infectious diseases which continue to plague global health. The nearer future will show whether nanoparticle-based immunization platforms will translate into improved vaccines for human use.

## 8 Opinion: suggested pathway to create vaccine candidates by fusing antigenic proteins to a virus-like lumazine synthase nanoparticle

### 8.1 Modelling

**Fig. 10:**
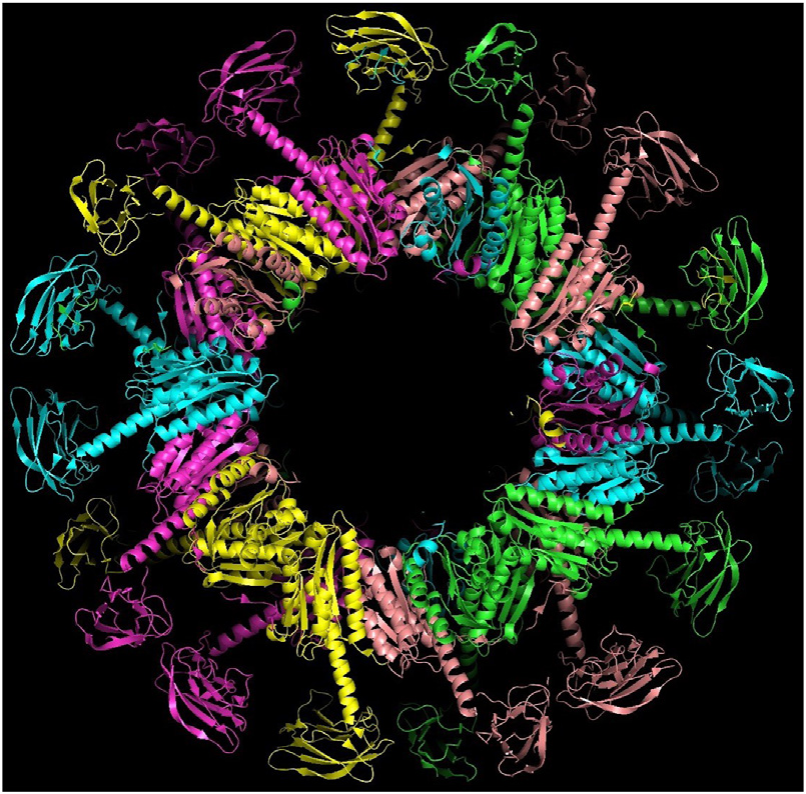
Cross section through the *Aquifex aeolicus* lumazine synthase capsid, T = 1, with a fusion of a Dengue virus spike protein E-DIII [70], PDB code 2R69. The Dengue virus antigens are fused to the C-termini of the lumazine synthase monomers, with help of a 10 residue Ala-Ala linker. The whole capsid hosts 60 spikes at its surface. The production of Fig. 9 by Prof. Luca Jovine, Karolinska Institutet, is greatly acknowledged.

1 Make use of structural data (PDB) of icosahedral *Aquifex aeolicus* lumazine synthase, T = 1, or T = 3, or use another suitable scaffold.
2 From structural coordinates (1HQK in PDB) extract a subunit pentamer
3 Fit linkers (Ala-Ala or Ala-Gly or Ala-Ser, may be 5–10 residues), by computer graphics, to the 5 C-termini (or 5 N-termini) at the outer pentamer surface. Probably it will be necessary to play with the linker length and –composition.
4 To the C-terminal end (N-terminal end) of the linkers fuse the N-terminus (or C-terminus) of a target viral or bacterial surface antigenic protein (or antigenic protein part), in such a way that the antigenic surface (the epitope) points into solvent space. Of course, a prior condition is that the atomic structure of the antigenic protein is known.
5 Rebuild the whole icosahedral structure (12 pentamers) of lumazine synthase with the fused epitopes from the single pentamer by proper application of the icosahedral symmetry operators.
6 Probably energy optimization will be needed to obtain a low energy model. This can be performed as rigid body refinement on a single pentamer, with the refinement shifts symmetrically applied to the rest of the pentamers, (e.g. Rosetta Design [63] would be a suitable software, among others). The obtained model will be very useful for structural comparisons later (Fig. 10).

### 8.2 Recombinant technology

1 Express the single lumazine synthase subunit with the fused antigenic protein by recombinant technology in a suitable prokaryotic or eukaryotic cell culture system. In case of a glycosylated antigenic fusion protein, mammalian cell culture has to be used. The protein can in this case be purified by lectin chromatography.
2 Chromatographic methods (e.g. gel chromatography) can show whether icosahedral nanoparticles have formed by self-assembly in the cell extract.
3 Electron microscopic images, using negative staining, or cryo electron microscopy with image reconstruction will show, in comparison with the structural model above, whether nanoparticles containing a dense array of fusion proteins have formed.

### 8.3 Encoding immunogen nanoparticles by RNA replicons

RNA replicons can serve as platforms for vaccine development [58]. The study by Melo et al. favours replicon delivery of designed immunogens as a practicable route for vaccine development. RNA replicons can express self-assembling nanoparticle immunogens. It has been shown that encapsulation in lipid nanoparticles resulted in effective antibody production in mice after i.m. administration.

### 8.4 Pre-clinical tests

Using suitable animal models, will subsequently be needed to show whether the obtained vaccine candidate is able to elicit broadly neutralizing antibodies.

## CRediT authorship contribution statement

**Rudolf Ladenstein:** Conceptualization, Investigation, Writing - original draft, Supervision, Funding acquisition, Writing - review & editing. **Ekaterina Morgunova:** Writing - review & editing, Visualization.

## Declaration of Competing Interest

The authors declare that they have no known competing financial interests or personal relationships that could have appeared to influence the work reported in this paper.
